# Supplementary material for: Patterns of Intron Gain and Loss in Fungi
Source: PLoS Biol. 2004 Nov 30;2(12):e422. doi: 10.1371/journal.pbio.0020422 (PMC532390; doi:10.1371/journal.pbio.0020422)
Supplement: Table S1 — Also available at http://genes.mit.edu/NielsenEtAl/. (4.3 MB ZIP). [file pbio.0020422.st001.zip › NielsenEtAl/html/1180.html]

AN5519.1.NCU09461.1.MG02434.1.FG06007.1


```
 CLUSTAL W (1.82) Multiple Sequence Alignments - Introns Inserted


Sequence 1: MG02434.1	187 aa
Sequence 2: FG06007.1	189 aa
Sequence 3: NCU09461.1	194 aa
Sequence 4: AN5519.1	185 aa
Alignment Length: 195 aa
Number Identitical Residues: 107 aa
Alignment Score (without introns) 5002


MG02434.1 	MINAFLVFNGQGQPRLTKFYTQL0ETNMQQRLISEIFTLVSNRPTGSCNFLPLPPLLAAS
NCU09461.1	MINAFLVFNGQGQPRLTKFYTQL0DTSIQQRLISEIFTLVANRPKGSCNFLPLPPLLAAS
FG06007.1 	MINAFLVFNGQGQPRLTKFYTQL0ETSIQQRLISEIFTLVSNRPAGSCNFLPLPPMLAAS
AN5519.1  	MINAVLVFNNNGQPRLTKFYTQI0DTQTKQSLIAQIYDLVAQRPPTACNFLPLPPILAR-
          	****.****.:***********: :*. :* **::*: **::**  :********:**  

MG02434.1 	GTSHTDSTEQNDVPSLVTYRNYATLYFIVISTATESPLALIDLIQVYVEALDKLFENVCE
NCU09461.1	STSTSSSEPHNDVPSLVTYRNYATLYFIIISTSTESPLALIDLIQVYVESLDRLFENVCE
FG06007.1 	GTSHTSSEEQNDVPTLVTYRNYATLYFIVISTSTESPLALIDLIQVYVEALDKLFENVCE
AN5519.1  	GASSSASTGPSDAPTQITYRTYATLSFIMISTSTESPLALIDLIQVFVEALDRIFENVCE
          	.:* : *   .*.*: :***.**** **:***:*************:**:**::******

MG02434.1 	LDLIFNFETLHSTLSEMIVGGVVVETNLDRIVAGVRAQ-GTVAKRQTNDGRGGGGGIGSG
NCU09461.1	LDLIFNFETLHATLGEMIVGGVVIETNMEKIVQGVRAQ-GTVAKRPVNESRGGGMGLGSG
FG06007.1 	LDLIFNFETLHSTLSEMIIGGVVIETNLDRIVSGVKAQ-GTVAKRPVNEGRGPTG-LGSG
AN5519.1  	LDLIFGFETMHAVLSEMIVGGVVVETNIDKIVAGVQSQEGSLGKKRAIQAASSGVGRG--
          	*****.***:*:.*.***:****:***:::** **::*.*::.*: . :. .   . *  

MG02434.1 	L----GIG---VWHGR
NCU09461.1	LGAGLGMGGNFVWSGR
FG06007.1 	L----GMGANFAWSGR
AN5519.1  	-----GLSGLAAWR--
          	     *:..  .*
```
